# Temporomandibular Disorders among Dutch Adolescents: Prevalence and Biological, Psychological, and Social Risk Indicators

**DOI:** 10.1155/2018/5053709

**Published:** 2018-04-17

**Authors:** Carolina Marpaung, Frank Lobbezoo, Maurits K. A. van Selms

**Affiliations:** ^1^Department of Oral Kinesiology, Academic Centre for Dentistry Amsterdam (ACTA), University of Amsterdam and Vrije Universiteit Amsterdam, Amsterdam, Netherlands; ^2^Department of Prosthodontics, Faculty of Dentistry, Trisakti University, Jakarta, Indonesia

## Abstract

**Aims:**

To assess the prevalence rates of pain-related temporomandibular disorders (TMDs) and temporomandibular joint (TMJ) sounds in a large group of Dutch adolescents, aged between 12 and 18 years and to determine if the same biological, psychological, and social risk indicators are related to both TMD pain and TMJ sounds.

**Methods:**

In this cross-sectional questionnaire survey, 4,235 questionnaires were analyzed, with an about equal gender distribution.

**Results:**

The overall prevalence of pain-related TMDs was 21.6% (26.1% for girls and 17.6% for boys) and that of TMJ sounds was 15.5% (19.3% for girls and 11.7% for boys). Logistic regression analyses revealed that the following variables appeared to be the strongest predictors of TMD pain: female gender, increasing age, sleep bruxism, biting on lips and/or cheeks, stress, and feeling sad. Regarding self-reported TMJ sounds, the multiple regression model revealed that female gender, increasing age, awake bruxism, and biting on lips and/or cheeks were the strongest predictors.

**Conclusions:**

TMDs are a common finding among Dutch adolescents. Except for the psychological factors that appeared to be associated with TMD pain only, pain-related TMDs and TMJ sounds shared similar biological risk indicators.

## 1. Introduction

Temporomandibular disorders (TMDs) is a collective term that embraces a variety of temporomandibular joint (TMJ) disorders, masticatory muscle disorders, headache disorders, and disorders affecting the associated structures [1, 2]. One way of classifying the different types of TMDs is by dividing them into two broad categories: (1) pain-related TMDs and (2) intra-articular TMDs [2]. Regarding the first category, pain can originate from the TMJs, but more frequently, the masticatory muscles are involved [3, 4]. Pain-related TMDs are usually transient over time and resolve without serious long-term effects [5, 6]. Intra-articular TMDs are expressed by biomechanical signs like TMJ sounds (clicking and crepitation), jaw locking, and limited mouth opening [2]. TMJ sounds are the most common expression of intra-articular TMDs [7] and usually occur without pain or jaw movement limitation [8, 9]. Even though both categories of TMDs are primarily present among young and middle-aged adults [10, 11], studies performed on children and adolescents seem to indicate that the prevalence of pain-related forms of TMDs increases with increasing age in this age group [11–13]. Likewise, several studies on intra-articular TMDs report an increase of TMJ sounds in the young population [8, 14, 15].

It is generally believed that a variety of biological, psychological, and social factors may reduce the adaptive capacity of the masticatory system, thus resulting in TMDs [6, 16]. Since pain-related TMDs and TMJ sounds represent clusters of related disorders in the masticatory system [6], this would imply that overlap exists among the risk indicators for both categories of TMDs. For instance, it is commonly believed that teeth grinding or jaw clenching (i.e., bruxism) causes TMD pain due to overloading of the musculoskeletal structures [17]. At the same time, bruxism-induced overloading of the TMJs that exceeds the normal adaptive capacity might result in more TMJ sounds due to degenerative changes of the anatomical structures, or a tendency of the disc to be dislodged off the condyle [18, 19]. Surprisingly, many risk assessment studies on TMDs in the young population focused on one category of TMDs only (e.g., [8, 13, 20]), whereas in others, the various signs and symptoms of TMDs were merged into one overall TMD diagnosis (e.g., [21–23]). As it is, however, generally agreed that TMDs represent a nonspecific umbrella term, it is essential to differentiate pain-related TMDs from intra-articular TMDs. The aims of the present study, therefore, were (1) to assess the prevalence rates of self-reported pain-related TMDs and TMJ sounds in a large group of adolescents aged between 12 and 18 years, (2) to determine their associations with biological, psychological, and social risk indicators, and (3) to determine if the same risk indicators are related to both categories of TMDs.

## 2. Materials and Methods

### 2.1. Data Collection

This investigation was designed as a cross-sectional, population-based study. During three subsequent semesters, participants were drawn from among adolescents attending nine Dutch secondary schools that were willing to participate in this investigation. Because of time demand or other priorities at that time, 23 schools declined participation. All approached schools were dispersed over the southern and western parts of Netherlands and were situated in urban areas. Prior to the data collection, the parents/legal representatives received an information letter about the study. The children and/or the parents/legal representatives had the right to refuse participation.

On the day of data collection, a questionnaire was handed over to the schools' pupils and collected several minutes later, before the lessons started. This questionnaire contained 17 items that covered demographic items, sleep and awake bruxism, signs and symptoms of TMDs, and psychosocial and behavioural factors [24]. Most questions were derived from already existing questionnaires, like the Dutch translation of the Research Diagnostic Criteria for Temporomandibular Disorders (RDC/TMD) [25] and an oral habits questionnaire [26]. During the time the questionnaires were completed, the pupils were supervised by the class teacher and the investigators to ensure that the questionnaires were completed individually. Due to this approach, the participation rate was 100%. The institutional review board of the Academic Centre for Dentistry Amsterdam (ACTA) and the school boards of the participating schools approved the data collection procedures. Prior to the investigation, the feasibility of the research process was field-tested in a pilot study. In addition, the test-retest reliability of the employed questionnaire was assessed, yielding fair-to-good to excellent reliability scores. For detailed information about the data collection methods, see van Selms et al. [24].

### 2.2. Outcome Variables

- Orofacial pain, indicative of TMD pain, was assessed by means of the following question: “Have you had pain in the face, jaw, temple, in front of the ear or in the ear?” (no, yes). The question referred to the presence of pain within the last month.
- The presence of TMJ sounds was assessed using the question “Does your jaw make a clicking or popping sound when you open or close your mouth, or while chewing?” (no, yes). The question referred to the presence of TMJ sounds within the last month.

Since no clinical diagnoses were established in this study, the term “pain-related TMDs” has to be interpreted as “pains indicative of TMD pain” and “TMJ sounds” as “self-perceived TMJ sounds.”

### 2.3. Independent Variables

#### 2.3.1. Biological Items

- Age (years) and sex (0, “male”; 1, “female”).
- The presence of sleep bruxism was assessed using the question “Have you been told, or did you notice yourself, that you grind your teeth or clench your jaws when you are asleep?” The presence of awake bruxism was assessed using the question “Do you grind your teeth or clench your jaws during the day?” These questions referred to the last month, and the pupils could choose between no, yes, or unknown. Other oral activities that may be stressful to the masticatory system were asked by the following four questions: Do you chew on chewing gum? Do you bite your nails? Do you bite on pens/pencils? Do you bite your lips/cheeks? Again, these questions referred to the last month, and the answer possibilities were no, occasionally, regularly, often, and very often.
- The following exogenous aspects were assessed: “Do you smoke cigarettes?” and “Do you drink alcohol?” (both questions: no, occasionally, regularly, often, and very often).

#### 2.3.2. Psychological Items

- An impression of the psychological status was assessed by means of the following two questions “Are you stressed?” and “Are you feeling sad?” (both questions referred to the last month: no, occasionally, regularly, often, and very often).

#### 2.3.3. Social Items

- Ethnic background was classified following the method of Statistics Netherlands (CBS), using the country of birth from both parents. This procedure resulted in a classification into two subgroups, namely, native Dutch (i.e., both parents were born in Netherlands, regardless of the country of birth of the subject; coded “0”) and nonnative Dutch (i.e., all other subjects; coded “1”).
- Educational level was characterized by the type of the secondary educational system that was followed. Depending on their abilities, Dutch children around the age of 12 can choose for either vmbo, vmbo/havo, havo, havo/vwo, or vwo. The vmbo diploma gives access to advanced vocational education, the havo diploma to polytechnic education, and the vwo diploma to university education. The 5-point Likert scale item educational level was recoded into a dichotomous variable (vwo (1) versus the other levels (0)).

### 2.4. Data Analysis

Descriptive statistics included frequency distributions of each of the independent variables. In order to determine the prevalence rates of TMD pain and TMJ sounds, the prevalence data were stratified by gender and age and ratios were calculated. The chi-square test was performed to test the association between TMD pain and TMJ sounds as depicted in a 2 × 2 contingency table. To determine the association between the outcome variables and each of the independent variables, hierarchical logistic regression analyses were performed. First, single regression analyses were executed to determine the associations between each of the various predictors and the outcome variable. Regarding the ordinal variables, initial analyses were based on the full range of the 5-point Likert response options, and linearity of their effect on the presence of TMD pain was checked by analysis of dummy variables. When the regression coefficients of the dummy variables consistently increased or decreased, linearity was considered present. In case of a nonlinear association, the variable was dichotomized. Second, independent variables that showed at least a moderate association with the outcome measure were entered in a multiple regression model. Due to the fact that the large sample size may impact the corresponding *P* values, a more conservative level of significance was chosen (i.e., *P* value < 0.05 instead of *P* value < 0.1). Subsequently, the variables with the weakest association with the outcome variable were removed from the multiple regression model. This was repeated in a backward stepwise manner until all variables that were retained in the model showed a *P* value < 0.01; for each removed independent variable, the P-to-Exit is reported. Of the independent variables included in the final model, the odds ratios and their confidence intervals are reported. All analyses were conducted using the IBM SPSS Statistics 24 software package (IBM Corp., Armonk, NY). The data in the multiple regression model were checked for multicollinearity, using a tolerance value < 0.10 and a variance inflation factor > 10.

## 3. Results

Initially, a total of 4,285 pupils, with ages ranging from 10 to 22 years, completed the questionnaire. Since the present study focuses on TMD pain during adolescence, the data of pupils under twelve years (children) and above eighteen years (adults) were excluded (*n*=42; <1% of the total number). An additional eyeball verification of the paper questionnaires was performed in order to check the face validity of the data. In case a pupil deliberately had noted only extremes on all single items, this questionnaire was removed from further analysis (*n*=8). Therefore, the final sample consisted of 4,235 adolescents with a mean age of 14.5 (±1.6) years (Table 1). Of the 3,940 adolescents who completed the question about gender, 1,966 (49.9%) were girls. In addition, 82.0% of the adolescents were classified as native Dutch, and 43.7% of the pupils followed the highest educational level (vwo).

**Figure 1:**
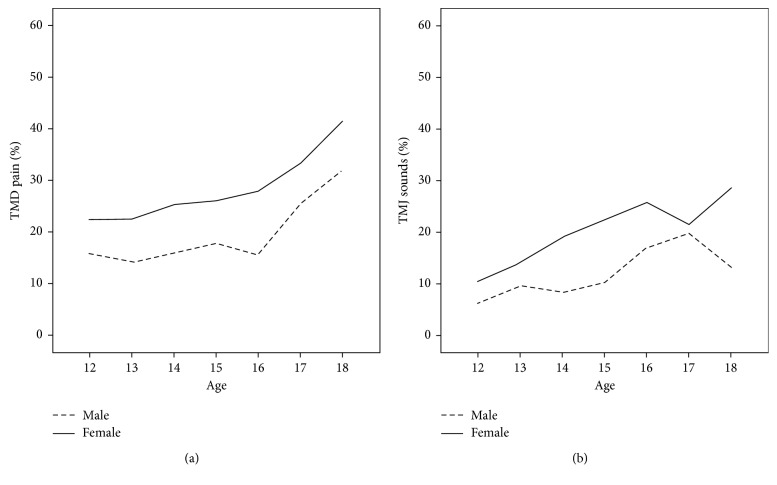
Age- and gender-specific prevalence of TMD pain (a) and TMJ sounds (b) among Dutch adolescents.

Of the 3,935 adolescents who completed the questions about gender and TMDs, the overall prevalence of pain-related TMDs was 21.6% (26.1% for girls and 17.6% for boys). The overall prevalence of TMJ sounds was 15.5% (*n*=3,920; 19.3% for girls and 11.7% for boys). The prevalence rates of both TMD pain and TMJ sounds, stratified by age and gender, revealed that girls had higher rates at all ages studied and that the prevalence tended to increase with age for both genders (Figure 1). TMD pain and TMJ sounds appeared to be highly associated (*χ*^2^(1) = 176.6; *P* < 0.001).

In order to find out which biological, psychological, or social factors had the strongest association with the presence of pain-related TMDs, logistic regression analyses were performed. In the first step, all variables were entered consecutively in a single regression model in order to determine their unadjusted association with the TMD pain. Regarding the included 5-point ordinal variables, inspection of the regression coefficients of the dummy variables revealed that perfect linearity of their effect on the presence of TMD pain was present only for the predictor “biting lips and/or cheeks.” All ordinal variables were therefore dichotomized (no = 0; all other categories = 1).  Table 2 shows the results of the single and multiple regression models. Except for the biological items gum chewing and nail biting and the social items ethnic background and educational level, all variables had a significant association with TMD pain in the single regression model. According to the multiple regression model, the following variables appeared to be the strongest predictors of TMD pain: female gender, increasing age, sleep bruxism, biting on lips and/or cheeks, stress, and feeling blue. There were no signs of multicollinearity among the predictor variables in the final model.

Table 3 shows the results of the regression analyses with the presence of self-reported TMJ sounds as an outcome variable. Again, most biological items were associated with joint sounds in the single regression model. In addition, feeling stressed and feeling sad had a significant association with TMJ sounds. The multiple regression model revealed that female gender, increasing age, awake bruxism, and biting on lips and/or cheeks were the strongest predictors of TMJ sounds.

## 4. Discussion

The present questionnaire study aimed to assess the prevalence rates of two categories of temporomandibular disorders (TMDs), namely, pain-related manifestations of TMDs and TMJ sounds, in a large group of Dutch adolescents aged between 12 and 18 years. In addition, we examined which biological, psychological, or social risk indicators were associated with them and if both categories of TMDs yielded similar risk indicators. The results demonstrated that self-reported TMD pain is relatively common among 12- to 18-year-old Dutch adolescents, with an overall prevalence of about 20%. Besides the fact that the occurrence of TMD pain was highly associated with that of TMJ sounds, this pain was correlated to female gender, increasing age, reports of sleep bruxism, biting on lips and/or cheeks, stress, and feeling sad. The overall prevalence of TMJ sounds was about 15%; female gender, increasing age, awake bruxism, and biting on lips and/or cheeks were the best predictors. Except for the psychological factors that appeared to be associated with TMD pain only, pain-related TMDs and TMJ sounds shared similar biological risk indicators.

### 4.1. Prevalence of TMD Pain

It is generally acknowledged that depending on the study, the prevalence of TMD pain in children and adolescents varies widely [27]. In 2007, a large-scale study was published that focused on TMD pain among adolescents aged 12–19 years [13]. Of the 28,899 adolescents that participated, 4.2% reported TMD pain during their annual routine examination in Public Dental Service (PDS) clinics. In another Swedish study, seven percent of the 862 adolescents from a public dental clinic were diagnosed with TMD pain [28]. This rate was also found in a recent study on Norwegian adolescents [29]. The most likely explanations for the fact that the present study yielded a higher prevalence rate (namely, 21.6%) are differences in diagnostic criteria and the method of data collection. In the present study, orofacial pain had to be present within the last month, whereas in the study by Nilsson, a time span of one week was used. In the studies by List et al. and Ostensjo et al., a clinical pain diagnosis according to the Research Diagnostic Criteria for Temporomandibular Disorders (RDC/TMD) was set, which may have resulted in a lower prevalence. On the other hand, when these clinical criteria were applied in two Brazilian studies performed on young adolescents, it was concluded that about 25% of the schoolchildren could be diagnosed with painful TMDs [20, 30]. As long as no uniform diagnostic criteria are available to obtain a reliable diagnosis of TMDs in the young population, studies on this topic will continue to present a multitude of different results. Future studies must therefore aim to develop a standardized assessment tool for the young population. Unfortunately, the recently published Diagnostic Criteria for TMD (DC/TMD) [2] have not yet been validated for usage among children and adolescents.

### 4.2. Risk Indicators for Pain-Related TMDs

Regarding the role of biological risk indicators on pain-related forms of TMDs, we demonstrated that the prevalence of TMD pain increases with increasing age in the period of adolescence. This is in line with several other studies (e.g., [11, 13, 31, 32]) and coincides with the suggestion that pubertal development increases the probability of self-reported TMD pain [12, 31]. Moreover, girls had higher rates of TMD pain at all ages studied compared to boys (namely, 26.1% and 17.6%, resp.), which corroborates with most studies on this topic (e.g., [12, 13, 28, 33]). Even though it is likely that sex differences exist in basic pain mechanisms and in associated psychosocial factors, the mechanisms underlying this difference are still not well understood [34]. Another biological factor that is frequently suggested to be associated with TMD pain in adolescents is overloading of the masticatory system due to oral habits (e.g., [20, 35]). As a result, it was not surprising that the final regression model included sleep bruxism and the adverse oral habit “biting on lips and/or cheeks.”

Based on the present findings, it appeared that the two included psychological factors (namely, being stressed and feeling sad) contributed significantly to the presence of TMD pain among adolescents. Again, this is not surprising as both factors are frequently mentioned in relation to this pain (e.g., [31, 36–38]). The same neurotransmitters, especially serotonin and norepinephrine, are involved in both pain and mood regulation [39]. An increase of cortisol secretion in people with high psychological load has also been shown to be related with chronic pain development [40]. However, caution has to be paid to this assumption, as causal links have not been clearly defined. Do these factors increase the risk of TMD pain or are they the result of this pain because such persons have become more stressed and less cheerful by their pain condition?

Finally, the social factors ethnic background and educational level were not associated with the presence of TMD pain. The negative findings in this study might show that differences in ethnicity and educational level in Dutch adolescents do not necessarily represent different social environments in relation to the report of pain. Out of a vast range of social factors that have been considered to influence an individual's pain behaviour, parent emotions, behaviours, and health seem to play an important role in a child's pain experience [41]. This topic might be an interesting avenue for future research.

### 4.3. Prevalence of TMJ Sounds

The overall prevalence of self-reported TMJ sounds was 15.5%, which is in line with approximately 14% as reported in a recent meta-analysis on the prevalence of TMJ sounds (click or crepitation) in children and adolescents [42]. Unfortunately, the authors of that systematic review did not differentiate between boys and girls. The gender-specific prevalence rates that we found in the present study (19.3% for girls and 11.7% for boys) seem to corroborate with those presented in earlier studies [12, 43]. On the other hand, even though a lower overall prevalence was found in a study of Feteih (8.7% of the participants reported joint sounds), they still observed a higher prevalence in girls [44]. It is generally acknowledged that differences in methodology lead to considerable variation in prevalence of TMJ sounds [42]. However, it can still be concluded that TMJ sounds are a commonly reported sign of TMDs in the adolescent population.

### 4.4. Risk Indicators for TMJ Sounds

As for TMD pain, four biological factors appeared to be associated with TMJ sounds. Consistent with other studies on the young population, the prevalence of TMJ sounds increased considerably with age [14, 15, 45], especially during adolescence. Until now, there is no explanation for this trend. It has been suggested that increasing age leads to a temporary space insufficiency within the TMJ [14]. During the period of adolescence, the articular eminence gets its more prominent anatomical shape [46], which can cause a lack of space within the TMJ complex [14]. As a result, this insufficient space forces the disc to be pushed from its normal position on top of the condyle to the anterior or anterolateral side during the closing movement of the mouth. The disc only resumes its normal position during the opening movement, during which TMJ sounds are produced [47, 48]. As for TMD pain, female gender was found to be associated with TMJ sounds. However, conflicting evidence exists regarding this association [14, 15, 49]. As the current study utilized self-reported data, the finding that prevalence rates are higher among girls might also be due to the fact that female adolescents report physical symptoms more often than their male counterparts [50–52]. Finally, the associations found in this study between daytime clenching and/or grinding and TMJ sounds and between biting on lips and/or cheeks and TMJ sounds corroborate with other studies [53–55]. A possible explanation for these associations is that adverse oral activities cause compression of the articular disc as was shown in a finite element model study [56]. The occurring stresses may facilitate the disc to be dislodged off the head of the condyle to the anterior or anterolateral side, thus creating clicking sounds upon condyle translation movements [18, 47].

### 4.5. Methodology

This study has several limitations. First of all, pain-related TMDs and TMJ sounds were obtained by a questionnaire with no objective confirmation of signs and symptoms, thus being at risk of recall bias. However, high validity can exist between self-reported pain questions and the outcome of a clinical examination in adolescents [13]. Likewise, in a longitudinal study on signs and symptoms of TMDs in Finnish adolescents by Könönen and Nystrom, reported and clinically examined TMJ clicking sounds correlated significantly with each other [45]. Second, for an indication of TMJ sounds, all pupils had to note if they experienced any clicking or popping sound when opening or closing the mouth. The presence of crepitation was, however, not asked for. Even though crepitation has a much lower occurrence in the adolescent population, if present at all [15, 42], other results might have been obtained in case this type of TMJ sound was included. Third, the present study was conducted in an adolescent population composed of nonpatients. However, to fulfill the objective of determining associations with biological, psychological, and social risk indicators, different results might have been obtained in case a group of symptomatic patients was included. Therefore, further studies should be performed with representative samples of patients with TMD pain and TMJ sounds as well. The fourth aspect that should be mentioned is that, with increasing age, larger cognitive capacity, and better recall, older adolescents might remember and therefore report any physical symptoms better than younger ones [50]. This might have influenced the obtained results with respect to prevalence and associations.

## 5. Conclusions

This study indicates that both pain-related manifestations of TMDs and TMJ sounds are a common finding in the adolescent population. Both categories share similar biological risk indicators, whereas psychological factors were only associated with pain-related TMDs.

## Figures and Tables

**Table 1:** Descriptive statistics of the predictor variables.

Independent variable	
Age (years)	14.5 (±1.6)
Gender	
Male	1,974 (50.1%)
Female	1,966 (49.9%)
Sleep bruxism	
No	2,874 (82.0%)
Yes	633 (18.0%)
Awake bruxism	
No	3,334 (90.0%)
Yes	372 (10.0%)
Chewing gum	
No	261 (6.2%)
Yes	3,943 (93.8%)
Biting nails	
No	2,105 (50.0%)
Yes	2,104 (50.0%)
Biting pens and pencils	
No	2,397 (56.9%)
Yes	1,819 (43.1%)
Biting lips and/or cheeks	
No	1,793 (42.6%)
Yes	2,414 (57.4%)
Smoking cigarettes	
No	3,658 (86.7%)
Yes	559 (13.3%)
Alcohol consumption	
No	2,166 (51.4%)
Yes	2,046 (48.6%)
Being stressed	
No	1,680 (39.9%)
Yes	2,534 (60.1%)
Feeling sad	
No	2,183 (51.8%)
Yes	2,030 (48.2%)
School type	
Lower levels	2,386 (56.3%)
Highest level	1,849 (43.7%)
Ethnic background	
Native Dutch	3,368 (82.0%)
Nonnative Dutch	740 (18.0%)

The dichotomized categorical variables are presented as absolute numbers (ratio); age is presented as mean value (±standard deviation).

**Table 2:** Single and multiple logistic regression models for the prediction of TMD pain among Dutch adolescents.

	Single regression				P-to-Exit	Multiple regression (*n*=3,131)		
	*n*	*P* value	OR	95% CI		*P* value	OR	95% CI
Biological items								
Female gender	1,964	<0.001	1.66	1.42–1.94		0.008	1.29	1.07–1.55
Age (years)	4,106	<0.001	1.12	1.06–1.17		<0.001	1.11	1.05–1.17
Smoking cigarettes (positive)	559	<0.001	1.60	1.31–1.95	0.467	—	—	—
Drinking alcohol (positive)	2,044	<0.001	1.49	1.29–1.73	0.097	—	—	—
Sleep bruxism (positive)	631	<0.001	1.76	1.45–2.14		<0.001	1.60	1.29–1.98
Awake bruxism (positive)	372	<0.001	1.93	1.53–2.44	0.262	—	—	—
Chewing gum (positive)	3,938	n.s.	1.00	0.74–1.36				
Biting nails (positive)	2,100	n.s.	0.95	0.82–1.10				
Biting pencils (positive)	1,816	<0.001	1.34	1.16–1.55	0.435	—	—	—
Biting lips and/or cheeks (positive)	2,409	<0.001	1.69	1.45–1.97		0.003	1.33	1.10–1.61
Psychological items								
Being stressed (positive)	1,679	<0.001	2.33	1.97–2.74		<0.001	1.60	1.28–1.99
Feeling sad (positive)	2,025	<0.001	2.14	1.84–2.48		<0.001	1.55	1.27–1.88
Social items								
Non-Dutch ethnicity	738	n.s.	0.97	0.80–1.18				
Highest educational level	1,848	n.s.	1.03	0.88–1.19				

Associations are expressed as odds ratio (OR) and 95% confidence interval (CI). For each removed predictor variable, the P-to-Exit is reported; n.s. = not significant. Significance levels are 0.05 and 0.01, respectively.

**Table 3:** Single and multiple logistic regression models for the prediction of TMJ sounds among Dutch adolescents.

	Single regression				P-to-Exit	Multiple regression (*n*=3,337)		
	*n*	*P* value	OR	95% CI		*P* value	OR	95% CI
Biological items								
Female gender	1,959	<0.001	1.81	1.51–2.16		<0.001	1.77	1.45–2.16
Age (years)	4,090	<0.001	1.19	1.13–1.26		<0.001	1.21	1.14–1.29
Smoking cigarettes (positive)	557	<0.001	1.55	1.23–1.94	0.156	—	—	—
Drinking alcohol (positive)	2,040	<0.001	1.53	1.29–1.82	0.406	—	—	—
Sleep bruxism (positive)	633	<0.001	1.62	1.30–2.02	0.045	—	—	—
Awake bruxism (positive)	369	<0.001	1.98	1.53–2.56	0.262	<0.001	1.79	1.36–2.36
Chewing gum (positive)	3,922	0.046	1.50	1.01–2.22	0.011	—	—	—
Biting nails (positive)	2,093	n.s.	1.14	0.96–1.34				
Biting pencils (positive)	1,811	n.s.	1.34	0.96–1.35	0.435	—	—	—
Biting lips and/or cheeks (positive)	2,406	<0.001	1.66	1.39–1.98		<0.001	1.46	1.19–1.80
Psychological items								
Being stressed (positive)	1,668	<0.001	1.81	1.50–2.17	0.042	—	—	—
Feeling sad (positive)	2,019	0.001	1.31	1.12–1.56	0.123	—	—	—
Social items								
Non-Dutch ethnicity	732	n.s.	0.81	0.64–1.02				
Highest educational level	1,846	n.s.	0.86	0.73–1.02				

Associations are expressed as odds ratio (OR) and 95% confidence interval (CI). For each removed predictor variable, the P-to-Exit is reported; n.s. = not significant. Significance levels are 0.05 and 0.01, respectively.

## Data Availability

All relevant data are within the paper. On request, the data sets generated and/or analyzed during the current study are available from the corresponding author.
